# Individual and national financial impacts of informal caring for people with mental illness in Australia, projected to 2030

**DOI:** 10.1192/bjo.2022.540

**Published:** 2022-07-18

**Authors:** Deborah Schofield, Melanie J. B. Zeppel, Robert Tanton, Jacob Lennert Veerman, Simon J. Kelly, Megan E. Passey, Rupendra N. Shrestha

**Affiliations:** Centre for Economic Impacts of Genomic Medicine (GenIMPACT), Macquarie Business School, Macquarie University, Sydney, New South Wales, Australia; Centre for Economic Impacts of Genomic Medicine (GenIMPACT), Macquarie Business School, Macquarie University, Sydney, New South Wales, Australia; National Centre for Social and Economic Modelling, University of Canberra, Australian Capital Territory, Australia; School of Medicine, Griffith University, Gold Coast, Queensland, Australia; National Centre for Social and Economic Modelling, University of Canberra, Australian Capital Territory, Australia; University Centre for Rural Health, University of Sydney, Lismore, New South Wales, Australia; Centre for Economic Impacts of Genomic Medicine (GenIMPACT), Macquarie Business School, Macquarie University, Sydney, New South Wales, Australia

**Keywords:** Carers, labour force participation, welfare payments, lost income, mental illness

## Abstract

**Background:**

Mental illness has a significant impact not only on patients, but also on their carers’ capacity to work.

**Aims:**

To estimate the costs associated with lost labour force participation due to the provision of informal care for people with mental illness in Australia, such as income loss for carers and lost tax revenue and increased welfare payments for government, from 2015 to 2030.

**Method:**

The output data of a microsimulation model Care&WorkMOD were analysed to project the financial costs of informal care for people with mental illness, from 2015 to 2030. Care&WorkMOD is a population-representative microsimulation model of the Australian population aged between 15 and 64 years, built using the Australian Bureau of Statistics Surveys of Disability, Ageing and Carers data and the data from other population-representative microsimulation models.

**Results:**

The total annual national loss of income for all carers due to caring for someone with mental illness was projected to rise from AU$451 million (£219.6 million) in 2015 to AU$645 million (£314 million) in 2030 in real terms. For the government, the total annual lost tax revenue was projected to rise from AU$121 million (£58.9 million) in 2015 to AU$170 million (£82.8 million) in 2030 and welfare payments to increase from AU$170 million (£82.8 million) to AU$220 million (£107 million) in 2030.

**Conclusions:**

The costs associated with lost labour force participation due to the provision of informal care for people with mental illness are projected to increase for both carers and government, with a widening income gap between informal carers and employed non-carers, putting carers at risk of increased inequality.

Mental illness is reported to be responsible for as much as 32% of years lived with disability (YLDs) and 13% of disability-adjusted life-years (DALYs), according to the Global Burden of Disease Study.^1^ This makes mental illness the chronic condition with the largest disease burden in terms of YLDs and the condition that accounts for a similar proportion of DALYs as cardiovascular and circulatory diseases.^1^ It causes the most disability in the UK, representing about 28% of the national disease burden.^2^ In Australia, healthcare spending on mental illness in 2015–2016 was estimated at AU$9 billion.^3^ There are also financial burdens on individuals and the government associated with reduced income during working life and retirement.^4,5^

Mental illness has a significant impact not only on the person with the illness but also on the informal carers caring for them.^6^ Provision of care can be a significant burden to informal carers, affecting both their psychological^7^ and financial well-being.^8^ A Spanish study analysing the socioeconomic costs of mental illness showed substantial financial costs of informal care, with the cost contributing to about 18% of the costs associated with mental illness.^9^ In Australia, the annual cost of provision of informal care in 2015 was estimated at AU$13.2 billion.^10^ The reduced capacity for informal carers to work can also lead to feelings of isolation and impacts on their own mental health. One study from Australia which surveyed 225 carers reported that the caring role and issues associated with job inflexibility had an impact on the carers’ ability to work and also their own health.^11^ Other studies also report that lack of employment can result in financial pressure, as well as isolation, along with stress and anxiety.^11–13^

This study projects the financial costs in Australia of reduced employment due to caring for someone with a mental illness to 2030. Previous studies have estimated the impacts for only 1 year, whereas this study quantifies costs every 5 years from 2015 to 2030, including estimating costs such as income loss for carers and reduced tax and additional welfare payments for government.

## Method

**Fig. 1:**
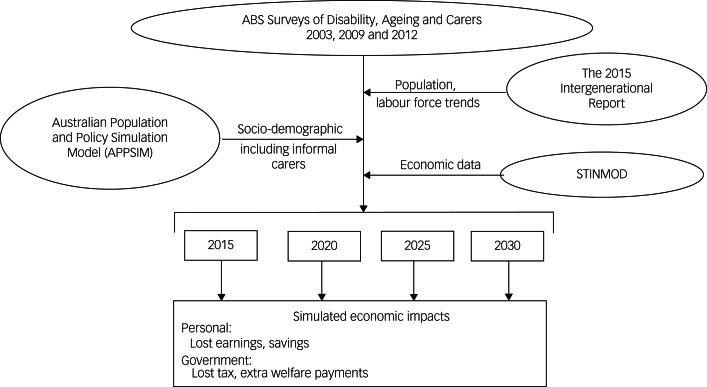
Schematic diagram of the Care&WorkMOD microsimulation model. ABS, Australian Bureau of Statistics; STINMOD, Static Incomes Model.

This study used the output data-sets of an Australian microsimulation model Care&WorkMOD. It was designed to project the financial costs of reduced capacity to work due to provision of care every 5 years from 2015 to 2030. Details of the model, including the data, are available elsewhere^14^ and Fig. 1 illustrates the structure of Care&WorkMOD.

The base data of Care&WorkMOD were person-level data for those aged 15 to 64 years in the Australian Bureau of Statistics (ABS) Surveys of Disability, Ageing and Carers (SDACs) in 2003, 2009 and 2012.^15,16^ In addition, the model consists of three other Australian population-representative data-sets: (a) forecasts of labour force participation and population size and demographic from the 2015 Intergenerational Report;^17^ (b) the sociodemographic projections, including that related to informal carers, from the Australian Population and Policy Simulation Model (APPSIM);^18^ and (c) output data-sets from the Static Incomes Model (STINMOD).^19^

Since the age-specific incidence of mental illness has been reported to be around the same level over time in Australia^20^ and globally and any increase in overall population prevalence has been reported to be mainly due to demographic change,^21^ no change in age-specific future prevalence rates of mental illness was assumed in our model. The model accounted for a natural growth in mental illness cases due to demographic change – mainly the ageing population and population increase. This was done by statically ageing the three 2003, 2009 and 2012 SDAC data-sets to 2015 to 2030 at 5-yearly intervals. Static ageing reweights the data-set by modifying the survey weights assigned to each record to account for socioeconomic changes, including carer numbers, with the assumption that carers have the same propensity to be a carer over the projection period of 2015 to 2030. A reweighting algorithm GREGWT, based on the generalised regression method, was used for static ageing.^22^

Additional income, tax and government benefits information were imputed onto Care&WorkMOD from STINMOD for 2015 using synthetic matching.^23^ The economic data from STINMOD were indexed to produce projections to 2030. Earned income and taxes were assumed to increase in real terms at 1% per year, the same percentage as used by the Australian Treasury.^24^ Informal carers who are not working may be entitled to receive Carer Payments and individuals who are unable to work because of their own illness or disability may be eligible to receive the Disability Support Pension from the Australian Government as welfare payments. Both these welfare payments also have 1% real growth in accordance with the current government indexation rates. The government policy for other government benefits is indexation using Consumer Price Index growth, which results in zero real growth.^24^

Our analysis focused on the financial burden to primary carers of people with mental illness. A primary informal carer is defined as a person who provides the most informal assistance for at least 6 months without payment for the provision of care. In the SDACs, informal carers were asked about the chronic health conditions the person they were caring for had and about the main condition among these. Primary informal carers who reported the main reason they were not in the labour force as ‘Someone else's ill health or disability’ and who also reported ‘a mental illness’ as the main condition the person they were caring for had were considered an informal carer out of the labour force owing to caring for someone with ‘mental illness’ in this analysis. Respondents who provided other responses as their main reason for being out of the labour force were excluded from the analysis. ‘Mental illness’ was defined as diseases that were grouped into ‘mental and behavioural disorders’ by the ABS, which included depression/mood/affective disorders, dementia, schizophrenia, nervous tension/stress, phobic and anxiety disorders and other mental and behavioural disorders.

### Statistical analysis

The financial outcomes such as income, taxes and welfare payments were summarised using mean, standard deviation and median. The differences in financial costs for those not in the labour force (or lost productive life-years (PLY)) owing to care provision compared with those for people in the labour force (either full-time employed, part-time employed or unemployed) who were not providing care were estimated from counterfactuals using Monte Carlo methods. For each carer not in the labour force owing to caregiving, a counterfactual record of the same age group, gender and highest level of education was selected at random with replacement from the pool of non-carers who were in full-time or part-time employment or unemployed. The mean difference in the financial outcomes of carers not in the labour force owing to provision of care and their counterfactuals was estimated. Simulations were run for 5000 iterations, generating 5000 counterfactual data-sets. We report the average of the 5000 simulations along with the 95% confidence interval (CI), estimated using the percentile method. Similar analyses were undertaken to estimate the differences in financial outcomes of carers not in the labour force and people employed full-time and part-time who were not carers. Costs are reported in real 2015 Australian dollars.

We used SAS, version 9.4 for Windows (SAS Institute, Cary, NC, USA), to conduct the analysis. The Microdata Review Panel at the Australian Bureau of Statistics approved the use of ABS data in this study.

## Results

There were 389 records of primary informal carers, aged between 15 and 64 years, of people with mental illness in Care&WorkMOD. Once weighted, these records represent about 53 700 Australian primary informal carers, aged between 15 and 64 years, of people with mental illness in 2015, with the majority of carers (75%) being women. Primary informal carers for someone with mental illness were projected to increase to 63 200 in 2030, a 18% increase. Of these 389 records, 88 were for individuals who were out of the labour force because of their caring role. Once weighted, these data represent a population of approximately 12 900 Australian primary informal carers aged 15 to 64 years who were out of the labour force owing to caring for someone with a mental illness in 2015. The number of primary informal carers who were out of the labour force owing to provision of informal care of someone with a mental disorder was projected to increase to about 14 700 by 2030 (Table 1). The estimated proportion of different mental illnesses that primary informal carers were caring for and the estimated proportion of different mental illnesses among Australians who reported having them as a main chronic condition in 2015 are presented in supplementary Table 1, available at https://doi.org/10.1192/bjo.2022.540.

**Table 1:** Weekly total income, welfare payments and taxes paid by non-carers and by informal carers who were not in the labour force owing to caring for someone with mental illness (NILF carers), Australian population aged 15–64 years, in 2015 AU$

		2015				2020				2025				2030			
Labour force status	Survey records, *n*	Weighted pop.	Mean	s.d.	Median	Weighted pop.	Mean	s.d.	Median	Weighted pop.	Mean	s.d.	Median	Weighted pop.	Mean	s.d.	Median
Weekly total income (AU$) of individuals^a^																	
Employed full-time, non-carers	51 455	7 362 000	1544	1611	1280	8 002 000	1625	1677	1340	8 549 000	1727	1773	1410	9 028 000	1854	1820	1502
Employed part-time, non-carers	22 120	3 116 000	647	836	488	3 158 000	714	915	550	3 390 000	752	976	576	3 592 000	799	1041	623
NILF carers	88	12 900	374	228	392	13 400	378	233	376	13 500	392	238	433	14 700	406	232	464
Total weekly welfare payments (AU$)																	
Employed full-time, non-carers	51 455	7 362 000	26	81	0	8 002 000	26	80	0	8 549 000	27	83	0	9 028 000	27	83	0
Employed part-time, non-carers	22 120	3 116 000	80	142	6	3 158 000	81	142	6	3 390 000	81	141	6	3 592 000	80	140	6
NILF carers	88	12 900	343	203	360	13 400	335	203	360	13 500	336	204	360	14 700	342	198	360
Total weekly tax payments (AU$)																	
Employed full-time, non-carers	51 455	7 362 000	357	519	254	8 002 000	378	557	265	8 549 000	405	601	280	9 028 000	439	641	302
Employed part-time, non-carers	22 120	3 116 000	72	287	4	3 158 000	83	315	10	3 390 000	87	338	11	3 592 000	94	361	16
NILF carers	88	12 900	0	5	0	13 400	1	7	0	13 500	1	7	0	14 700	1	7	0

pop., population.

a. Includes both earned income and welfare payments.

Informal carers of someone with a mental illness who were out of the labour force owing to provision of care reported having about a quarter of the mean weekly income of people employed full-time who were not carers (about AU$374 (£182) *v*. AU$1544 (£751.80)) in 2015. By 2030, these carers were projected to receive about AU$406 (£197) per week compared with AU$1854 (£903) per week for full-time employed non-carers. Informal carers who were not in the labour force as a result of caring responsibilities, on average, received AU$343 (£167) per week in welfare payments, which were estimated to remain relatively constant to 2030 (Table 1). The estimated expenditure on welfare payments for carers was substantially greater than the welfare payments received by non-carers working both full-time and part-time.

The mean weekly income of full-time employed non-carers compared with those not in the labour force owing to caring for people with mental illness, after controlling for age, gender and highest level of education, was about $952 (95% CI $854–$1068) higher in 2015, with the difference expected to increase to $1156 (95% CI $1047–$1282) in 2030 (Table 2). The mean weekly income of part-time employed non-carers was estimated to be $339 (95% CI $269–$427) higher than those of carers of people with mental illness in 2015, with the difference increasing to $425 (95% CI $348–$523) in 2030. Carers who were out of the labour force owing to caring for people with mental illness were found to receive mean weekly welfare payments $297 (95% CI $285–$308) higher than non-carers employed full-time in 2015, increasing to $329 (95% CI $317–$339) in 2030 (Table 2).

**Table 2:** Differences in average weekly income, weekly welfare payments and weekly tax payments between non-carers employed full-time or part-time and informal carers who were not in the labour force owing to caring for someone with mental illness (NILF carers), Australian population aged 15–64 years, in 2015 AU$

	2015		2020		2025		2030	
	AU$ difference	95% CI	AU$ difference	95% CI	AU$ difference	95% CI	AU$ difference	95% CI
Difference in weekly total income								
Non-carers employed full-time *v*. NILF carers	952	(854–1068)	1006	(904–1125)	1089	(981;1223)	1156	(1047–1282)
Non-carers employed part-time *v*. NILF carers	339	(269–427)	357	(279–455)	396	(319–493)	425	(348–523)
Difference in weekly welfare payments received								
Non-carers employed full-time *v*. NILF carers	−297	(−308 to −285)	−298	(−309 to −286)	−310	(−320 to −298)	−329	(−339 to −317)
Non-carers employed part-time *v*. NILF carers	−245	(−262 to −226)	−248	(−265 to −230)	−259	(−277 to −241)	−276	(−293 to −258)
Difference in weekly tax payments								
Non-carers employed full-time *v*. NILF carers	258	(225 to 301)	270	(236 to 314)	291	(255 to 340)	309	(271 to 354)
Non-carers employed part-time *v*. non-carers NILF owing to caring for someone with mental illness	77	(58 to 105)	81	(60 to 110)	88	(67 to 117)	92	(72 to 118)

There was a substantial increase in the estimated financial costs of caring for someone with mental illness from 2015 to 2030. When aggregated at the national level, the total income loss due to caring for someone with mental illness was estimated at $450.9 million (95% CI $390.2–$521.9 million) in 2015, which was projected to increase to $645.4 million (95% CI $565.8–$735.8 million) in 2030, around a 43% increase (Table 3). The total cost due to lost tax revenue was also projected to grow substantially. Total annual lost income tax revenue due to reduced labour force participation of informal carers caring for someone with mental illness was estimated at $120.9 million (CI $101.7–$146.5 million) in 2015, increasing to $169.4 million (95% CI $143.8–$200.6 million) in 2030, while total welfare payments received by them were projected to increase from $169.7 million (95% CI $159.8–$179 million) in 2015 to $220.1 million (95% CI $209.3–$230.1 million) in 2030 (Table 3).

**Table 3:** Aggregated annual income loss of primary carers and income tax revenue loss and extra welfare payments for the government due to lost labour force participation of primary carers caring for someone with mental illness, Australian population aged 15–64 years, in 2015 AU$

Cost	2015		2020		2025		2030	
	AU$ million	95% CI	AU$ million	95% CI	AU$ million	95% CI	AU$ million	95% CI
Individuals: lost income	450.9	(390.2–521.9)	501.0	(433.9–581.1)	554.0	(481.0–636.8)	645.4	(565.8–735.8)
Government: lost tax payments	120.9	(101.7–146.5)	133.1	(112.2–161.2)	145.8	(122.7–174.8)	169.4	(143.8–200.6)
Government: increased welfare payments	169.7	(159.8–179.0)	179.1	(168.9–188.3)	189.1	(178.9–198.9)	220.1	(209.3–230.1)

## Discussion

In this study, we projected a substantial increase in the income lost to informal carers of persons with a mental illness from 2015 to 2030. As welfare payments are indexed to inflation (a lower rate than wages growth), there was projected to be less growth in welfare payments over the same period, meaning that the income gap between carers and non-carers is projected to increase. This will lead to an increase in inequality between non-carers and informal carers of people with mental illness. Impacts of compounding disadvantage are likely to be greatest for older women, who are most often informal carers, with reduced incomes and savings for retirement.^11,25^

Our results showed that there would be a substantial increase in the financial costs of caring for someone with mental illness, with the estimated aggregate loss of income increasing by about 43% over 15 years from 2015 to 2030. There are a number of reasons for this increase over time. These include increased participation of women in the labour force and higher rate of education, particularly among women, both of which are reflected in increased aggregate losses.

The results from this study suggest that, since a large proportion of informal carers of people with mental illness are out of the workforce, interventions that support carers or people with mental illness could assist those carers who are able to work to continue or return to some paid work, thereby reducing their economic disadvantage.^26^ Considerable evidence of financial hardship has been reported by carers of people with mental illness. Accordingly, there is an urgent need to review policies providing support to carers so that these better meet the needs of those who can return to work and those who may not be able to return to paid work.^11^ For example, New Zealand recently introduced an hourly payment for family carers who are not be able to return to work, at a cost of AU$32 (£15.6) million over 4 years.^27^ Currently, demand for mental health services outstrips supply but improving access to mental health services would improve mental health outcomes. This would support the capacity of informal carers to return to or remain in work, thus improving national productivity and economic outcomes.^28^

Both improved respite care and intervention strategies to prevent or treat mental illness could benefit the psychological and financial status of carers who wish to work. Increasing funding of intervention strategies that aim to prevent mental illness would also enable both carers and patients to increase workforce participation.^26^ For example, preventing depression through group therapy was estimated to prevent 5200 prevalent cases of depression and add 520 people to the labour force. This was estimated to increase private incomes by AU$19 (£9.3) million and tax revenues by AU$2.4 (£1.17) million and to reduce transfer payments by AU$2.6 (£1.26) million per year.^26^

Many informal carers of people with mental illness require respite care for the people they care for but are unable to access it, with adequate respite being recognised as an important unmet need.^29^ Regular respite care, along with policies enabling flexible work, are both strategies to enable informal carers to return to work.^30,31^ Previous studies have also reported that informal carers could be more supported by additional information about available services and provision of more support from such services.^11,30,32^

### Limitations

This study has several limitations. The data used are self-reported. However, this is a commonly used method recognised as having no more limitations than other methods of estimating labour force participation.^33^ Our study was limited to carers aged 15 to 64 years, caring for people in the same household. Results were based on 88 survey records of informal carers who were out of the labour force owing to caring for someone with mental illness. The study only focused on the main reason for being out of the labour force and did not capture secondary reasons. There may be some informal carers working part-time to enable them to provide care for someone with mental illness or working in a lower-paid full-time position to support their caregiving needs. Since there was no information in the SDAC about why the respondents were working part-time, we could not confirm this and estimate the financial costs associated with underemployment. However, we do know that carers are more likely to work part-time than those who do not have caring responsibilities. Carers caring for different mental illnesses may face different demands of caring that vary by the condition. For example, caring for someone with dementia, which in general is a progressive chronic condition, can be increasingly time-consuming compared with caring for someone with depression, which can be episodic in nature. However, owing to the small number of informal carers caring for specific mental illnesses, we have used the broader grouping of all informal carers caring for ‘mental and behavioural disorders’ in this analysis.

Our study data were collected prior to the COVID-19 pandemic and thus do not include the mental health effects of COVID-19 or strategies to contain the disease such as extended lockdowns. Further, the impact of COVID-19 on the Australian economy has not been taken into account, although the economic impacts for Australia have not been as great as for some other countries, with the Reserve Bank of Australia stating in June 2021 that ‘Australia's recovery has exceeded all expectations. Employment and output are already at pre-pandemic levels’.^34^ Australia's unemployment rate is currently 4.6%, with 5% traditionally considered full employment.^35^

## Data Availability

The data used in this study are from several different sources. Data from the Surveys of Disability, Ageing and Carers are available from the Australian Bureau of Statistics. Other data, such as Care&WorkMOD, STINMOD and APPSIM, are available from the corresponding author on reasonable request.

## Supplementary material

For supplementary material accompanying this paper visit http://doi.org/10.1192/bjo.2022.540. click here to view supplementary material
