# Supplementary material for: Individual and national financial impacts of informal caring for people with mental illness in Australia, projected to 2030
Source: BJPsych Open. 2022 Jul 18;8(4):e136. doi: 10.1192/bjo.2022.540 (PMC9345331; doi:10.1192/bjo.2022.540)
Supplement: Supplementary file 1 [file S2056472422005403sup001.docx]

Supplementary Table 1: Estimated proportion of different mental illness that informal carers who were not in the labour force due to caring for mental illness were caring for and the estimated proportion of different mental illness among Australians who reported having them as a main chronic condition in 2015

|  | Informal carers who were not in the labour force due to caring for someone with mental illness^1^ | | | Number of Australians with mental illness as a main condition^2^ | |
| --- | --- | --- | --- | --- | --- |
| Type of mental illness | Survey records | Weighted population | Proportion (%) | Weighted population | Proportion (%) |
| Dementia | 16 | 2,500 | 19.3 | 32,600 | 3.4 |
| Schizophrenia | 11 | 1,800 | 14.3 | 43,600 | 4.5 |
| Depression affective disorder (excluding postnatal depression) | 21 | 2,000 | 15.5 | 361,800 | 37.2 |
| Phobic and anxiety disorders | 8 | 1,000 | 7.4 | 315,600 | 32.4 |
| Nervous tension/stress | 10 | 1,400 | 10.7 | 169,400 | 17.4 |
| Other mental and behavioural disorders | 22 | 4,200 | 32.9 | 49,800 | 5.1 |
| Total | 88 | 12,900 | 100 | 972,800 | 100 |

^1^Source: Care&WorkMOD

^2^Source: ABS Survey of Disability, Ageing and Carers, 2015
